# Hair cell identity establishes labeled lines of directional mechanosensation

**DOI:** 10.1371/journal.pbio.2004404

**Published:** 2018-07-19

**Authors:** Marta Lozano-Ortega, Gema Valera, Yan Xiao, Adèle Faucherre, Hernán López-Schier

**Affiliations:** 1 Unit of Sensory Biology & Organogenesis, Helmholtz Zentrum München, Munich, Germany; 2 Centre for Genomic Regulation, Barcelona, Spain; National Institurte of Health, United States of America

## Abstract

Directional mechanoreception by hair cells is transmitted to the brain via afferent neurons to enable postural control and rheotaxis. Neuronal tuning to individual directions of mechanical flow occurs when each peripheral axon selectively synapses with multiple hair cells of identical planar polarization. How such mechanosensory labeled lines are established and maintained remains unsolved. Here, we use the zebrafish lateral line to reveal that asymmetric activity of the transcription factor Emx2 diversifies hair cell identity to instruct polarity-selective synaptogenesis. Unexpectedly, presynaptic scaffolds and coherent hair cell orientation are dispensable for synaptic selectivity, indicating that epithelial planar polarity and synaptic partner matching are separable. Moreover, regenerating axons recapitulate synapses with hair cells according to Emx2 expression but not global orientation. Our results identify a simple cellular algorithm that solves the selectivity task even in the presence of noise generated by the frequent receptor cell turnover. They also suggest that coupling connectivity patterns to cellular identity rather than polarity relaxes developmental and evolutionary constraints to innervation of organs with differing orientation.

## Author summary

Mechanosensory systems are essential for maintaining posture, gaze, and body orientation during locomotion. Such stability requires a coherent representation in the brain of the location and movement of mechanical stimuli. In fishes, mechanical stimuli at a given position activate direction-sensitive receptors called hair cells that are oriented with polarized directionality. These hair cells stimulate neurons that selectively connect with them based on polarity. We have addressed how neurons target hair cells based on polarity during development of the mechanosensory lateral line system in zebrafish. We show that neurons selectively connect based on the expression pattern of the transcription factor Emx2 in hair cells. We find that the lateral line can maintain directionality after damage and regeneration. Our data suggest a cellular mechanism that controls the formation, maintenance, and regeneration of labeled lines to enable directional mechanosensation.

## Introduction

**Fig 1:**
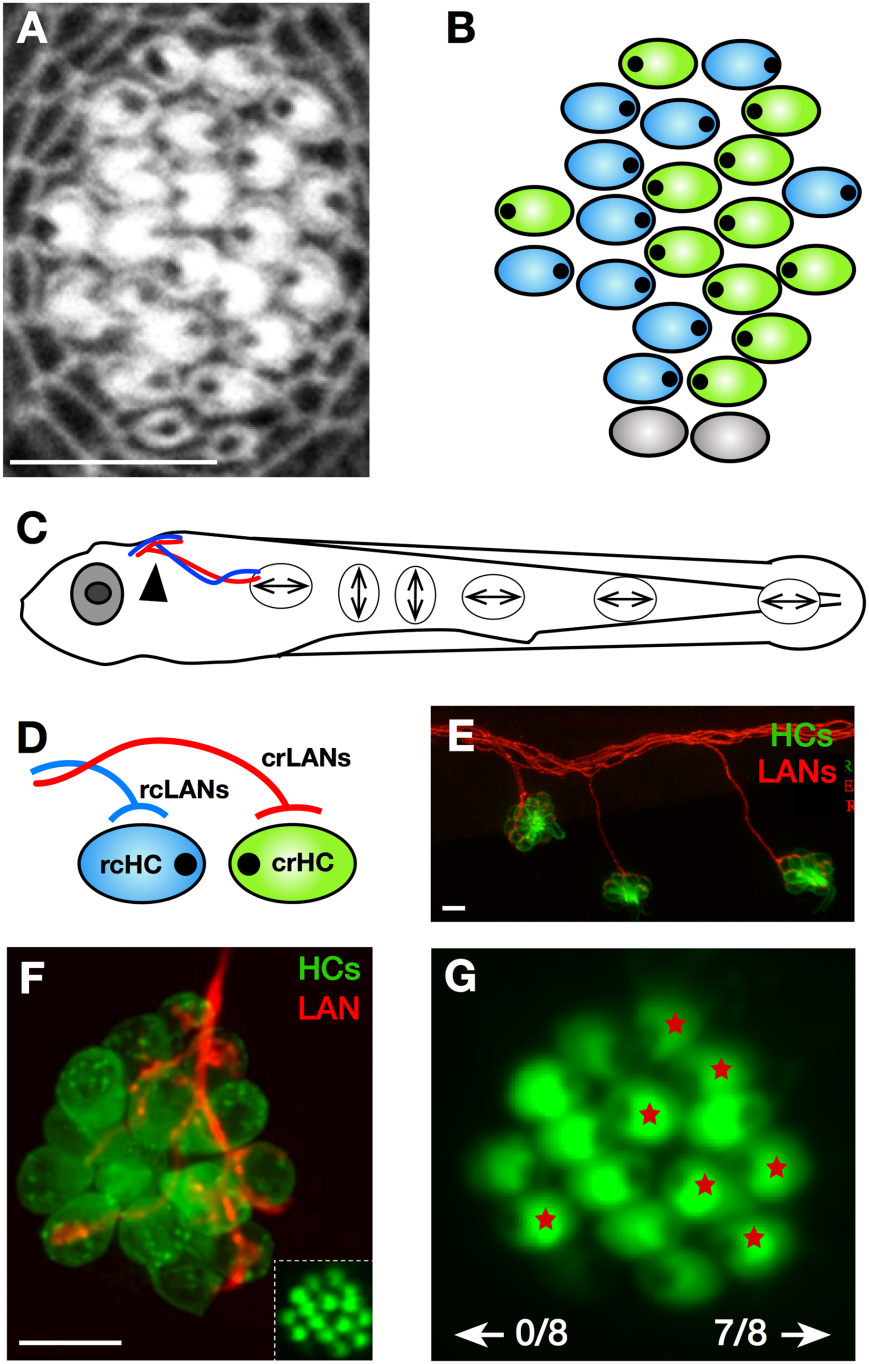
Architecture of the lateral line in larval zebrafish. (A–B) Apical aspect of a horizontal neuromast labeled with fluorescent phalloidin (panel A) and its schematic representation (panel B). These images highlight epithelial planar polarization. Hair cell orientation is indicated by an eccentric black dot. In panel B, blue hair cells are rcHCs and green are crHCs. Grey indicates a pair of newborn hair cells whose polarity is not yet evident. (C) Drawing of a larval zebrafish in which double-point arrows indicate the global orientation of horizontal and vertical neuromasts of the posterior lateral line. (D) Simplified scheme of the selective innervation of rcHCs (light blue) by rcLANs (blue) and crHCs (green) by crLANs (red). Polarity-selective synaptogenesis renders LANs tuned to the direction of mechanical stimulation of HCs, and form labeled lines of mechanosensory flow from the periphery to the brain. Each cell is meant to represent the entire population of identically oriented HCs in the neuromast and identically tuned LANs. (E) Confocal image of a lateral view of the trunk of a double-transgenic *Tg[pou4f3*:*GAP-GFP; hsp70l*:*mCherry-2*.*0cntnap2a]* larval zebrafish, showing LANs (red) and HCs (green). (F) Magnified frontal view of a neuromast of the transgenic line *Tg[myo6b*:*actb1-EGFP]* showing HCs (green). Inset at the bottom left shows the planar polarization of the hair cells. A single LAN (red) was marked by mosaic expression of mCherry. (G) Shows a higher magnification of the inset in panel F, in which the HCs that were innervated by the single mCherry-expressing LAN in panel F are marked with a red asterisk. In this example, 7 out of the 8 constituent crHCs were innervated by this identified LAN, and none of the rcHCs were synapsed by the same neuron. Scale bars are 10 μm. In all the panels and figures, dorsal is up and anterior is left. crHC, caudorostral HC; crLAN, caudorostral LAN; HC, hair cell; LAN, lateralis afferent neuron; rcHC, rostrocaudal HC; rcLAN, rostrocaudal LAN.

Two of the most highly conserved sensory-neural maps in vertebrates are those underlying postural control during locomotion and rheotaxis [1–8]. These sensory-motor processes originate from the action of mechanoreceptive hair cells residing, respectively, in the inner ear and in the lateral line [9–16]. Mechanosensitive ion channels in hair cells open or close according to the direction of deflection of the cells’ apical hair bundle [4, 17]. Consequently, the planar orientation of the hair bundles endows the sensory epithelium with directional excitability. The mammalian ear contains 3 vestibular receptive organs—the semicircular canals that detect angular shifts of head position, the sacculus that senses vertical movements, and the utricle that detects horizontal movements [18]. Utricular and saccular hair cells are divided into 2 groups of opposing hair bundle orientation, each situated across a line of polarity reversal [19, 20]. Similarly, the superficial lateral line in fishes senses velocity fields in the surrounding water [3, 6, 7, 13–15]. This system is formed by a collection of discrete mechanosensory organs called neuromasts, which are distributed across the body of the animal [11]. In zebrafish, each neuromast contains 16 to 20 mechanoreceptive hair cells that occur in 2 equally numbered populations with opposite planar polarity [21]. However, unlike the utricular and saccular epithelia, hair cells of both polarities intermingle in each neuromast (Fig 1A and 1B).

In addition to this local epithelial polarity, the global orientation of the hair cells varies according to the position of the neuromast. For example, the posterior lateral line of larval zebrafish contains neuromasts whose axis of planar polarity is oriented either parallel (horizontal neuromasts) or perpendicular (vertical neuromasts) to the anteroposterior body axis (Fig 1C) [21]. Horizontal neuromasts, therefore, are formed by hair cells that are activated by water flowing in the rostrocaudal direction (rcHCs) and by hair cells of caudorostral directional tuning (crHCs) (Fig 1D). Each branch of the lateral line in larval zebrafish contains around 60 bipolar lateralis afferent neurons (LANs) that are postsynaptic to the hair cells (Fig 1C–1E). Typically, each neuromast is innervated by the peripheral axons of 4 LANs, but each axon exclusively synapses with hair cells of identical planar polarity (Fig 1D–1G) [22, 23]. Because the transmission content of a sensory neuron is determined by all the inputs that it receives [24], planar-polarity–selective connectivity renders each LAN tuned to a single direction of mechanical stimulation to form a labeled-line sensory pathway from the periphery to the brain (Fig 1C and 1D).

How planar-polarity–selective synapses are established remains unknown [5, 8, 22, 25]. Understanding this process is important because it will reveal the basic rules that direct the development of neural circuits that enable directional mechanosensation, as well as how they are maintained despite continuous turnover of receptor cells [26–31]. Previous work has led to two models to explain polarity-selective synaptic connectivity in the lateral line. The biased-selector model suggests that synaptogenesis is inherently promiscuous and that selectivity occurs by the continuous rectification of mismatched synapses through a biased interaction between hair cells and the axonal population [32]. By contrast, the scaffold model presents constitutive basal projections from the hair cells as short-range processes that provide instructive polarity-specific scaffolds to the axons [33]. This idea is attractive in its simplicity, but it is based exclusively on phenomenological descriptions that have not been experimentally tested. Although both models are consistent with most results, they do not satisfy some observations. For example, the biased-selector model is difficult to reconcile with the invariable ratio of half of the LANs innervating every identically polarized hair cell. Conversely, the scaffold model cannot explain the loss of selectivity by solitary axons. Furthermore, two model-independent basic aspects of the selectivity mechanism remain unsolved: first, whether synaptic connectivity is defined locally in each neuromast independently of its global orientation and second, whether axons synapse with hair cells of identical polarity regardless of their local orientation (synaptic fidelity) or with hair cells of a specific orientation (synaptic partner matching). This distinction is important because the rules underlying fidelity (stochastic) and partner matching (deterministic) are likely to be mechanistically different.

## Results

### Hair cell basal projections are dispensable for selective connectivity

**Fig 2:**
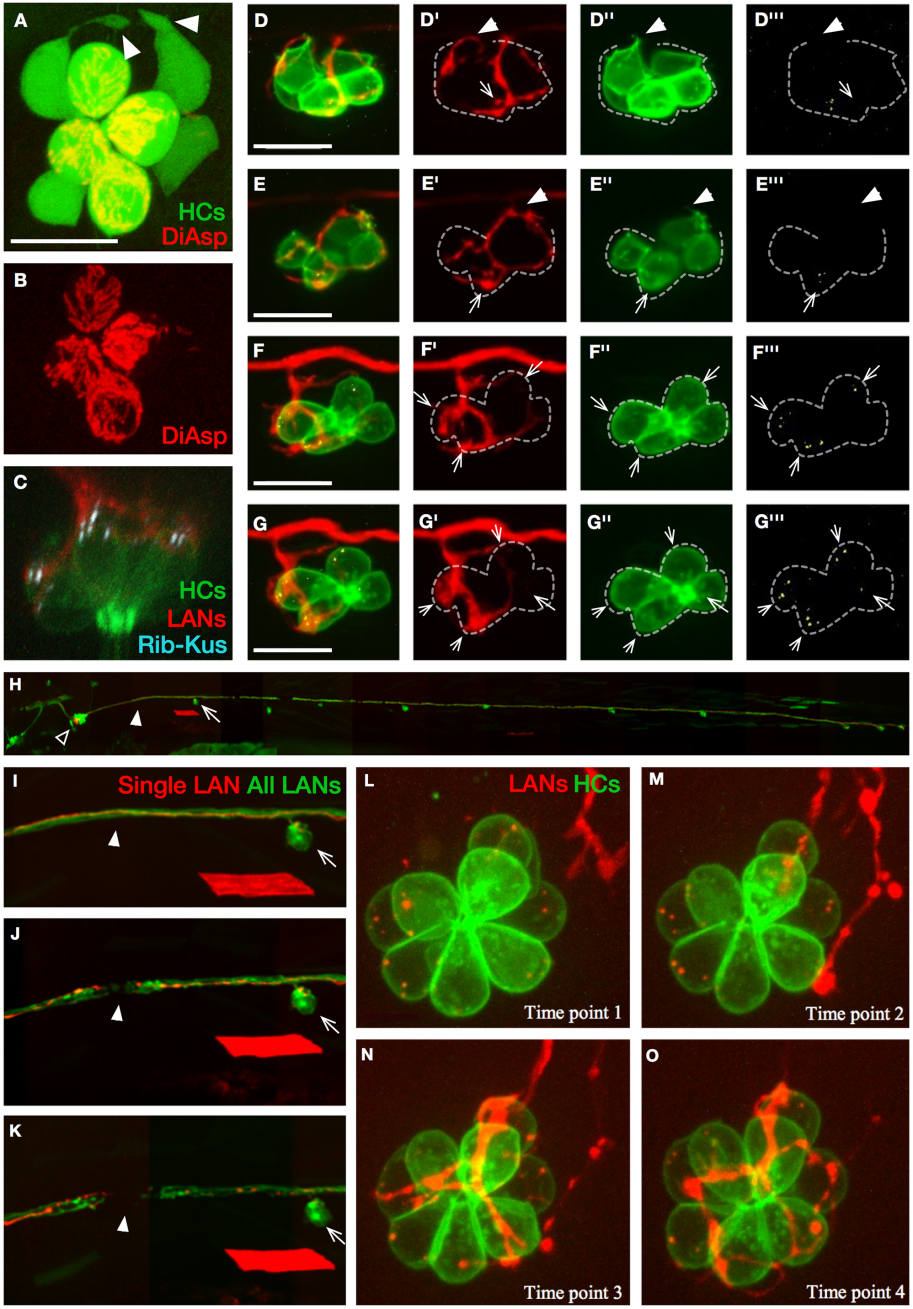
Live imaging of synaptogenesis in neuromasts. (A–B) Apical view of hair cells (green) in a horizontal neuromast of *Et(krt4*:*EGFP)sqet4* transgenics labeled with DiAsp (red), which only penetrates into mature hair cells through mechanotransducing channels. The image reveals basal projections (white arrowheads) in 2 of the 4 immature hair cells that are DiAsp(−) (white asterisks in panel B). (C) Confocal image of a neuromast in triple-transgenics *Tg[pou4f3*:*GAP-GFP; pou4f3*:*ctbp2l-mKOFP; nkhgn39d]*, showing juxtaposition of LAN neurites (red) with active-zone (Ribeye[+] puncta, light blue) in hair cells (green). (D–G’”) Selected time points from live imaging of innervation of hair cells (green) expressing Ribeye-Kusabira (light blue) by a singly marked LAN expressing mCherry (red), in double transgenics *Tg[pou4f3*:*GAP-GFP; pou4f3*:*ctbp2l-mKOFP]*. These panels were extracted from S1 Video. Ribeye(+) puncta are readily evident in hair cells (arrows) but absent from basal projections of hair cells (arrowheads in panel D–E’”). Synapses occur when LAN neurites and Ribeye(+) puncta are persistently juxtaposed over time (panel F–G’”). Dotted line outlines the hair cells. (H) Lateral view of a transgenic *Tg[nkhgn39d]* larval zebrafish expressing EGFP (green) in all LANs and mosaic expression of mCherry (red) in a single LAN, whose cell body (empty arrowhead) can be seen within the posterior lateralis ganglion. A neuromast (arrow) and the site of the eventual severing of the LAN peripheral axons (solid arrowheads) are indicated. The red rhomboid below the indicated neuromast is a singly marked myofiber resulting from nonspecific expression of mCherry in muscle. (I–K) Magnified views of the same fish in panel H, showing the LAN peripheral axons before laser-mediated severing (panel I), immediately after severing (panel J), and several hours after severing (panel K), evidencing the separation of the proximal and distal parts of the axon fragments. The arrows indicate the same neuromast and the solid arrowheads the site of the cut in every panel. The red rhomboid is the same myofiber in panel H. (L–O) Selected time points from live confocal imaging of regenerative innervation of mature hair cells (green) by the singly marked LAN (red). Panels were generated from S2 Video. Hair cells never produce basal projections during polarity-selective re-innervation. Scale bars are 10 μm in panel A, C, and D and 50 μm in panel I. DiAsp, 4-(4-diethy-laminostyryl)-N-methylpyridinium iodide; EGFP, enhanced green fluorescent protein; HC, hair cell; LAN, lateralis afferent neuron; Rib-Kus, Ribeye-Kusabira.

We began by experimentally testing the scaffold model using high-resolution live imaging in transgenic zebrafish expressing different fluorescent markers in LANs, hair cells, and their presynaptic active zone (Fig 2A–2C).

**Fig 3:**
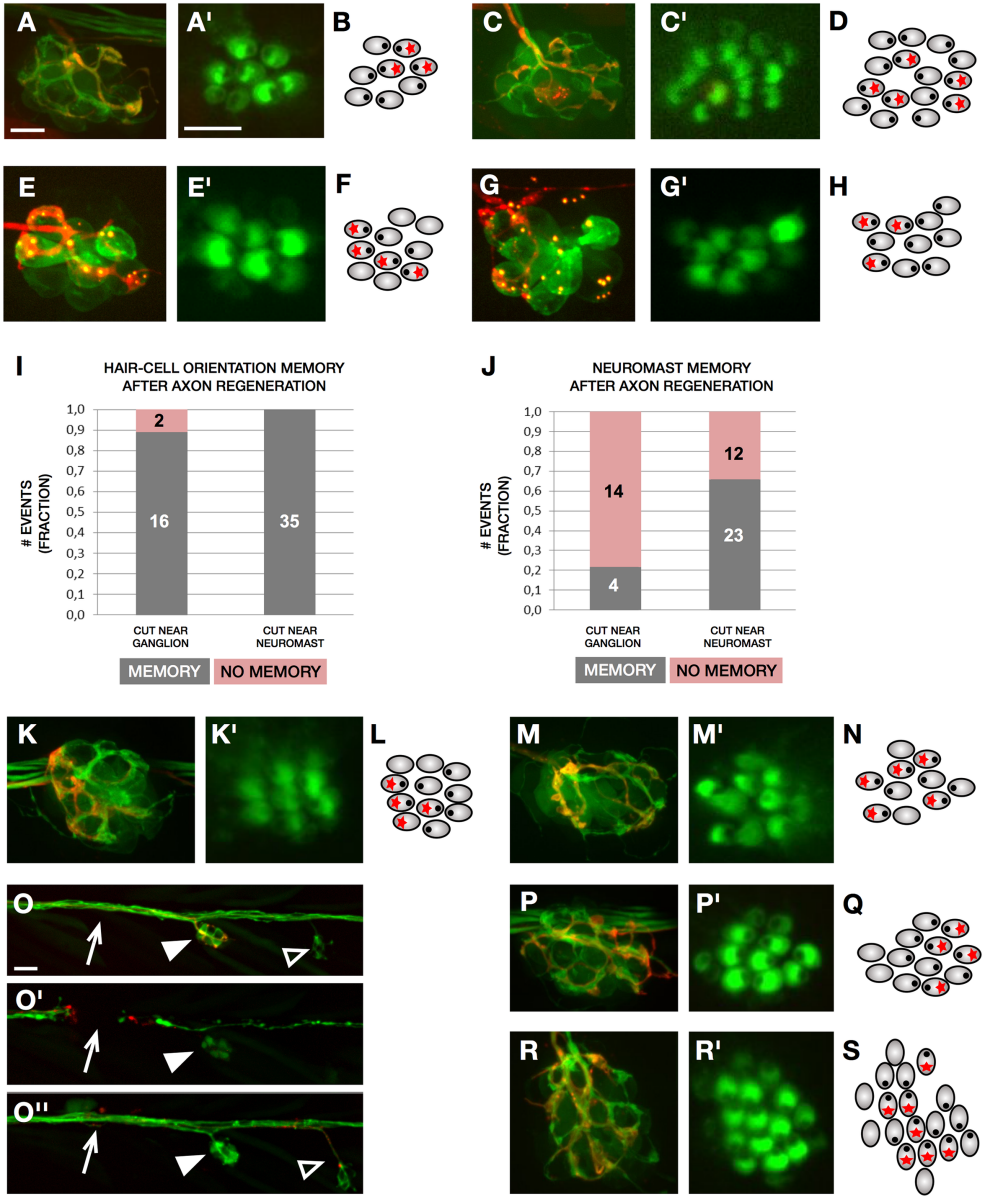
Regenerative synaptogenesis in neuromasts. (A–C) A neuromast of the transgenic line *Tg[myo6b*:*actb1-EGFP; nkhgn39d]* with mosaic expression of mCherry in a single LAN. (A) The individualized axon (red) synapses with crHCs. (A’) Magnified view of the hair bundles of hair cells in panel A. (B) Schematic representation of the example in panel A, in which hair cell orientation is indicated by an eccentric black dot and innervation by a red asterisk. (C) After severing, the individualized regenerated axon (red) recapitulate synapses with crHCs in the same neuromast. (C’) Magnified view of the hair bundles of hair cells in panel C. (D) Schematic representation of the example in panel C, indicating hair cell orientation (eccentric black dot) and synapses (red asterisk). (E) Neuromast double-transgenic *Tg[myo6b*:*actb1-EGFP; Ribeye-Kusabira]* showing a single LAN marked by mosaic expression of mCherry. The axon (red) synapses with rcHCs. (E’) Magnified view of the hair bundles of hair cells in panel E. (F) Schematic representation of the example in panel E, in which hair cell orientation is indicated by an eccentric black dot and innervation by a red asterisk. (G) After severing, the individualized axon (red) regenerated to recapitulate synapses with rcHCs. (G’) Magnified view of the hair bundles of hair cells in panel G. (H) Schematic representation of the example in panel G, indicating hair cell orientation (eccentric black dot) and synapses (red asterisk). (I) Quantification of hair cell innervation by regenerating singly marked axons of samples in which the axonal bundle was severed immediately below the neuromast (*N* = 35) (near neuromast) or furthest (*N* = 18) (near the posterior lateralis ganglion). All 35 axons cut near neuromast re-innervated hair cells of the original orientation—but 16 of the axons cut near the ganglion (0.89)—re-innervated the original orientation after regeneration (memory in grey), and 2 (0.11) did not (no memory in pink). (J) Quantification of neuromast innervation by regenerating singly marked axons of samples in which the lateralis nerves were severed immediately below the neuromast (near neuromast) (*N* = 35) or furthest (near the ganglion) (*N* = 18). A total of 23 (0.66) individualized axons cut near neuromast re-innervated the original neuromast (memory in grey), whereas 12 (0.34) re-innervated a different organ (no memory in pink). When axons were cut near the ganglion, 4 (0.22) re-innervated the original neuromast (memory in grey), and 14 (0.78) re-innervated a different organ (no memory in pink). (K–O”) Neuromast of the transgenic line *Tg[myo6b*:*actb1-EGFP; nkhgn39d]* with mosaic expression of mCherry in a single LAN. (K–K’) individualized axon (red) synapse with rcHCs. (K’) Magnified view of the hair bundles of hair cells in panel K. (L) Schematic representation of the example in panel K. (M) After axon severing and hair cell elimination, the individualized axon (red) regenerated to synapses with regenerated rcHCs in the same neuromast. (M’) Magnified view of the hair bundles of hair cells in panel M. (N) Schematic representation of the example in panel L–M. (O–O”) Selected time points from live confocal imaging of regenerative innervation of hair cells in the transgenic line *Tg[myo6b*:*actb1-EGFP; nkhgn39d]* (green) in an instance when a singly marked LAN (red) switched from a horizontal neuromast (solid arrowhead) to a vertical neuromast (empty arrowhead). Before laser-mediated severing (panel O), after severing (panel O’), and after regeneration (panel O”). The white arrow indicated the site of the cut. (P–P’, R–R’) Neuromast of the transgenic line *Tg[myo6b*:*actb1-EGFP; nkhgn39d]* with mosaic expression of mCherry in a single LAN. (P) The individualized axon (red) synapses with crHCs in a horizontal neuromast. (P’) Magnified view of the hair bundles of hair cells in panel P. (Q) Schematic representation of the example in panel P. (R) After severing, the individualized regenerated axon (red) switches to innervate a vertical neuromast, in which it synapses selectively with vdHCs. (R’) Magnified view of the hair bundles of hair cells in panel R. (S) Schematic representation of the example in panel R. Scale bars are 10 μm in A and A’ and 50 μm in O. crHC, caudorostral HC; HC, hair cell; LAN, lateral afferent neuron; rcHC, rostrocaudal HC; vdHC, ventrodorsal HC.

We first confirmed previous findings that only immature hair cells produce basal projections, up to 15 hours after they are born (Fig 2A and 2B) [33]. Synaptic connections between LANs and hair cells correlate with the stable juxtaposition of presynaptic puncta containing the active-zone constituent protein Ribeye and postsynaptic neurites (Fig 2C) [32, 34–36]. Next, we assessed synapses by individualized axons marked by mosaic expression of the red fluorescent protein mCherry in single LANs, in transgenic embryos co-expressing beta1-actin-enhanced green fluorescent protein (EGFP) and ctbp2a(Ribeye)-Kusabira in every hair cell (*Tg[myo6b*:*actb1-EGFP; pou4f3*:*ctbp2l-mKOFP]*) (Fig 2D–2G’” and S1 Video) [22, 26, 37, 38]. In less than one-half of the hair cells that were analyzed (*N* = 40), basal projections and axonal terminals physically interacted (16/40), but only a small minority of these interactions resulted in stable synapses (3/16). Moreover, although Ribeye(+) puncta were readily evident in hair cells (arrows in Fig 2D–2E’”), we could never detect them in basal projections (*N* = 31) (arrowheads in Fig 2D–2E’”, and S1 Video). These observations suggest that apposition of presynaptic projections and postsynaptic neurites correlates poorly with synaptic stabilization but does not rule out a function of these projections in synaptic selectivity. To test this possibility, we assayed synaptogenesis by regenerating axons. To this end, we severed axons using an ultraviolet laser delivered through the imaging objective (Fig 2H–2K) [39]. We found that cut axons regenerated to re-innervate preexisting mature hair cells that did not produce basal projections (Fig 2L–2O and S2 Video). To directly assess synaptic selectivity by regenerating axons, we marked single LANs with mCherry in the transgenic lines *Tg[myo6b*:*actb1-EGFP]* or *Tg[myo6b*:*actb1-EGFP; nkhgn39d]*, which reveal hair cell planar polarization in vivo (Fig 1F and 1G) [22, 40]. First, we corroborated that individually marked axons selectively synapse with hair cells of identical orientation before severing (Fig 3A, 3B, 3E and 3F).

Two days after severing, every regenerated axon selectively synapsed with identical-oriented hair cells despite the conspicuous absence of basal projections (*N* = 35) (Fig 3C, 3D, 3G and 3H). Together, these results indicate that, under our experimental conditions, presynaptic scaffolds do not form the bases of polarity-selective connectivity in the lateral line.

### Regenerating axons recreate synaptic partnership

During the course of the previous experiments, we made the surprising discovery that regenerating axons nearly always re-innervate hair cells not only of identical but also of the original planar orientation, recapitulating neuronal directional tuning (Fig 3I, right bar). In most of these cases, when axons were cut immediately below the neuromast, they also re-innervated the original neuromast (Fig 3J, right bar). Thus, we hypothesized that a potential memory of previous connectivity could rely on 2 possible sources of information: a cell-autonomous molecular code, or a nonautonomous cue that may include the neuronal extracellular basal lamina that remains around preexisting hair cells. Such nonautonomous blueprint of previous connectivity is reminiscent of the neuronal extracellular matrix that directs the repair of mammalian neuromuscular junctions after nerve crush and that is found in the piscine electric organs [41, 42]. To test this possibility, we cut axons and, subsequently, killed hair cells pharmacologically by incubating specimens in a solution of neomycin, which eliminates local sources of information about previous synapses. Yet this manipulation did not alter the recovery of LAN directional tuning after the concurrent regeneration of axons and hair cells (*N* = 8) (Fig 3K–3N). In a separate set of experiments, when axons were cut furthest from the neuromasts (near the lateralis ganglion), they often did not re-innervate the original neuromast (Fig 3J, left bar). Nevertheless, these axons formed selective synapses with identical-oriented hair cells in new neuromasts and almost always with those whose orientation was the same as the original (Fig 3I, left bar). Moreover, we found a remarkable correlation between horizontal and vertical neuromasts in that, when regenerating axons that originally innervated crHCs in horizontal neuromasts (Fig 3O–3Q) innervated a vertical neuromast, they always synapsed with ventrodorsal hair cells (vdHCs) (*N* = 4) (Fig 3O, 3O”, 3R and 3S). Conversely, rcHC-innervating LANs switched to re-innervate dorsoventral hair cells (dvHCs) in vertical neuromasts after regeneration (*N* = 3). Put together, these data reveal that LANs consistently recognize hair cells of identical polarity and of a specific orientation. These results are important because they suggest that hair cells of either orientation must have distinct marks that can be recognized by the axons during development and regeneration.

### Synaptic partnership occurs independently of coherent epithelial planar polarity

**Fig 4:**
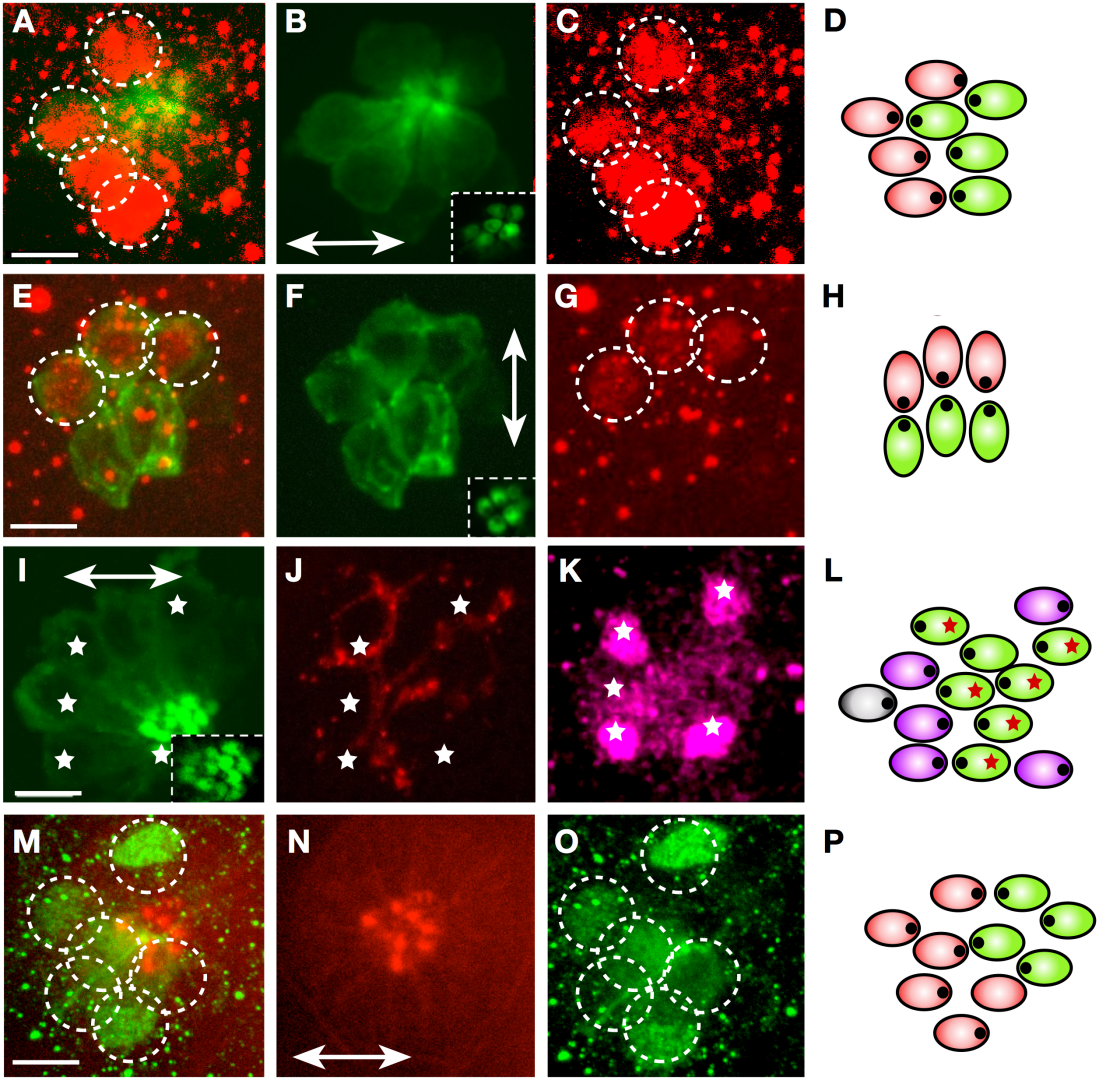
Emx2 expression in larval neuromasts. (A–C) Immunohistochemical staining of a horizontal neuromast in the transgenic line *Tg[myo6b*:*actb1-EGFP]* showing HCs (green) and Emx2 (red, dotted circles). Inset in panel B is a superficial image of the hair cells’ apices revealing their planar polarization. (D) Scheme of the neuromast in panel A–C indicating Emx2-expressing (red) and nonexpressing (green) hair cell. (E–G) Immunohistochemical staining of a vertical neuromast in the transgenic line *Tg[myo6b*:*actb1-EGFP]* showing HCs (green) and Emx2 expression (red, dotted circles). Inset in panel F is a superficial image of the hair cells’ planar polarization. (H) Scheme of the neuromast in panel E–G indicating Emx2-expressing (red) and nonexpressing (green) hair cell. (I–K) Immunohistochemical staining of a horizontal neuromast in the transgenic line *Tg[myo6b*:*actb1-EGFP]* showing HCs (green) (panel I), a single LAN expressing mCherry (red) (panel J). White asterisks indicate Emx2-expressing hair cells (purple) (panel K). Inset in panel I reveals hair cell planar polarization. (L) Scheme of the neuromast in panel I–K indicating Emx2-expressing hair cell (purple), nonexpressing hair cell (green), and the synapse of the individualized axon (red asterisks). The polarity of hair cell could not be determined (grey). In panel B, F, I, and N, neuromast orientation is indicated by a double-head arrow. (M–O) Staining of a horizontal neuromast in a *neurogenin1* mutant specimen, with an antibody to Emx2 and phalloidin to reveal hair cell apices. It shows Emx2-expressing cells (green, dotted circles) and hair cell orientation (panel N) (red, double-head arrow) (panel N). (P) Scheme of the neuromast in panel M–O indicating Emx2-expressing (red) and nonexpressing (green) hair cell, indicating hair cell orientation (eccentric black dots). Scale bars are 10 μm. HC, hair cell; LAN, lateralis afferent neuron.

It has been shown that planar-polarity–selective synapses occur independently of evoked activity [23, 32]. Therefore, we hypothesized that a cell-autonomous molecular cue differentiates hair cells of either orientation in each neuromast and identifies as “same” the horizontal rcHCs and vertical dvHCs, as well as the crHCs and vdHCs. While considering this possibility, we noted a previous study in mice showing that a mutation in the transcription factor Emx2 eliminates the line of hair cell polarity reversal in the utricular and saccular maculae without affecting coherent hair cell orientation, and that Emx2 is exclusively expressed on one side of this line in wild-type animals [19, 43]. A more recent report revealed that gain- and loss-of-function of Emx2 is sufficient to instruct hair cell orientation along a conserved planar polarity axis in zebrafish neuromasts [44]. These observations raise the possibility that Emx2 is part of a code underlying polarity-selective synaptogenesis. To test this hypothesis, we used a previously validated antibody to Emx2 to immunostain *Tg[myo6b*:*actb1-EGFP]* transgenic fish bearing individually labeled neurons. We first corroborated that Emx2-positive hair cells in wild-type fish were always rcHC and dvHC orientation, respectively, in horizontal and vertical neuromasts (Fig 4A–4H) [44].

Next, we found that each axon synapsed with either Emx2(+) or Emx2(−) hair cells (*N* = 6) (Fig 4I–4L and S5 Video). To test whether innervation determined the expression of Emx2 in hair cells, we examined zebrafish carrying a mutation in *neurogenin1*, which lack all innervation to the lateral line but have normal planar polarity in neuromasts [32]. We found that Emx2 was normally expressed in about half of the hair cells in mutant specimens (Fig 4M–4P).

**Fig 5:**
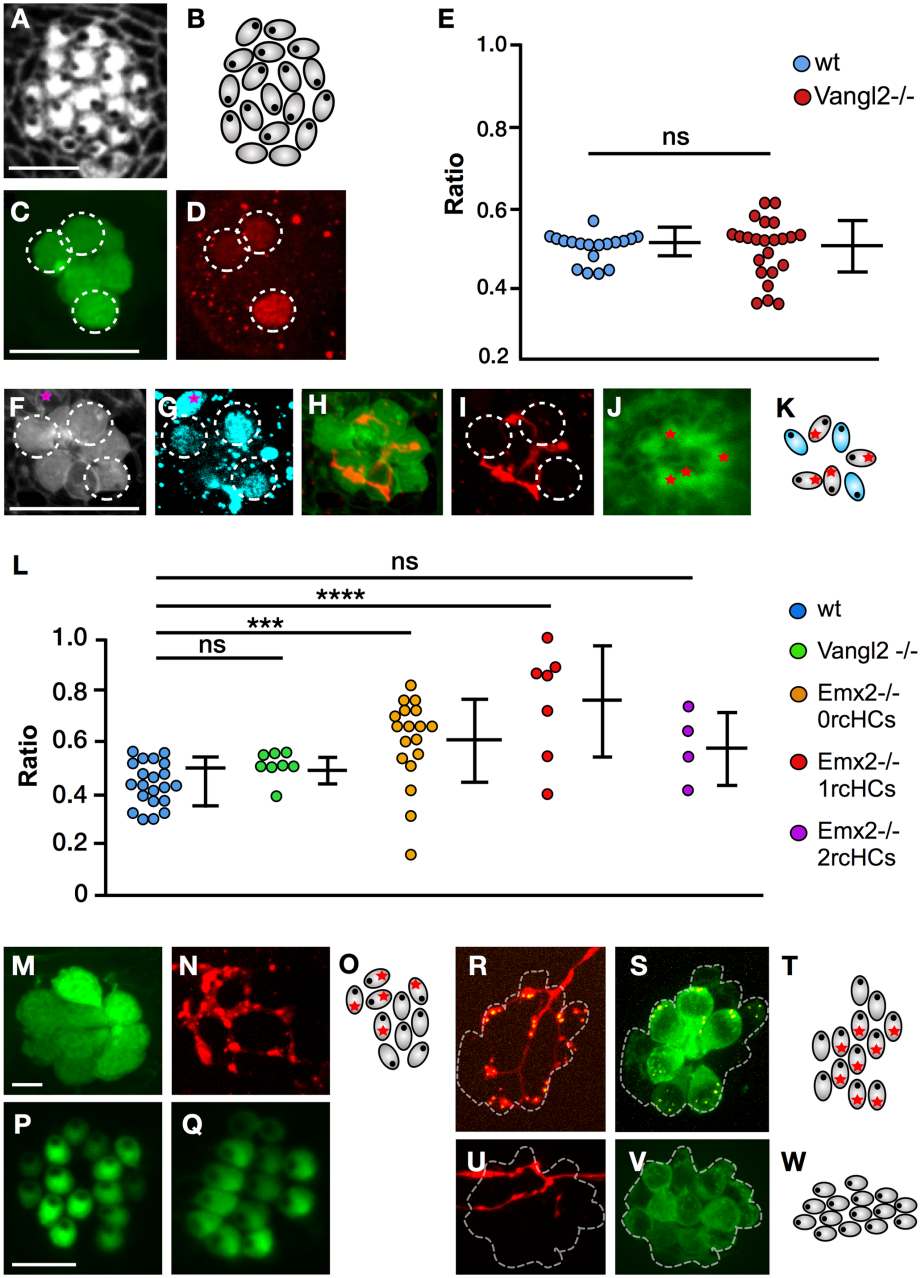
Regenerative synaptogenesis of neuromasts. (A–B) Apical aspect of a neuromast in Vangl2 mutant, labeled with fluorescent phalloidin (panel A) and its schematic representation (panel B). These images highlight the loss of coherent epithelial planar polarity in the neuromast. (C–D) Immunohistochemical staining of a horizontal neuromast in the transgenic line *Et(krt4*:*EGFP)sqet4* mutant for *Vangl2* showing HCs (panel C) and Emx2 (panel D). Dotted circles indicate Emx2(+) cells. (E) Quantification of Emx2 expression in wild-type (blue) and *Vangl2* mutant (red) neuromasts. Numbers on the y-axis indicate the fraction (ratio) of the hair cells expressing Emx2; one-way ANOVA. Error bars indicate mean ± SD. (F–K) A Vangl2−/− neuromast expressing EGFP in hair cells (panel F), immunostained for Emx2 (panel G), labeled with 488-phalloidin to reveal planar polarity (panel J) and expressing mCherry in a single LAN (panel H–I). Emx2 is expressed in 3 out of 7 hair cells (dashed circles) (panel G). A purple star marks an Emx2(+) cell that is not a hair cell (panel F–G). The marked LAN (panel H–I) innervates the 4 Emx2(−) hair cells as shown in the scheme (panel K), which is color-coded (blue for Emx2[+] and grey for Emx2[−]). (L) Quantification of the number of hair cells innervated by an identified axon in horizontal neuromasts in wild-type specimens (blue) and in those lacking Vangl2 (green) or Emx2 (orange/red/violet). Emx2 mutant neuromast were separated into those containing 0 rcHCs (orange), 1 rcHC (red), and 2 rcHCs (violet). Numbers on the y-axis indicate the fraction (ratio) of innervated hair cells. ***p < 0.001; one-way ANOVA. Error bars indicate mean ± SD. (M–N) A neuromast in the transgenic line *Et(krt4*:*EGFP)sqet4* expressing mCherry in a single LAN, showing HCs (panel M) and axonal terminal arborization (panel N). (O) A scheme of panel M–N indicating hair cell orientation (eccentric black dots) and the synapse of the individualized axon (red asterisks). (P–Q) A vertical (panel P) and horizontal (panel Q) neuromast from specimens lacking Emx2, showing the typical homogeneous polarization of hair cells. (R–S) A vertical neuromast lacking Emx2 in the transgenic line *Tg[pou4f3*:*GAP-GFP; pou4f3*:*ctbp2l-mKOFP]* expressing mCherry in a single LAN, showing juxtaposition of LAN neurites (red) with the active-zone Ribeye(+) puncta (yellow) (panel R), and presence of Ribeye(+) puncta (yellow) in hair cells (green) (panel S). Dotted line indicates the hair cells. (T) A scheme of panel R–S indicating hair cell orientation (eccentric black dots) and the synapse of the individualized axon (red asterisks). Note that the noninnervated hair cells are Ribeye(−) and therefore are likely immature. (U–V) A horizontal neuromast lacking Emx2 in the transgenic line *Tg[pou4f3*:*GAP-GFP]* expressing mCherry in a single LAN, showing no synapse of the LAN with any of the hair cells. Dotted line indicates the hair cells. (W) A scheme of panel U–V showing hair cell orientation (eccentric black dots). Scale bars are 10 μm. EGFP, enhanced green fluorescent protein; Emx2,; HC, hair cell; LAN, lateralis afferent neuron; ns, not significant; rcHC, rostrocaudal HC; Vangl2,; wt, wild-type.

The above results, together with the strict correlation between hair cell orientation and synaptogenesis, support the long-standing idea that synaptic selectivity and coherent epithelial planar polarity are mechanistically linked [33]. However, to examine this relationship experimentally, we took advantage of *trilobite* zebrafish, which harbors a loss-of-function mutation in the core planar-polarity protein van gogh-like 2 (Vangl2) [45] and displays a fully penetrant and strongly expressive randomization of hair cell orientation in the lateral line (Fig 5A and 5B) [46].

We reasoned that, if planar polarity and polarity-selective synaptogenesis are functionally linked, loss of coherent hair cell orientation will either induce neurons to innervate all, none, or random numbers of hair cells or to affect synaptic stability. First, we performed immunostaining for Emx2 in Vangl2 mutants and saw that, similarly to the wild type, approximately half of the hair cells in neuromasts were Emx2(+), indicating that neither the core planar-polarity pathway nor coherent hair cell orientation determine the expression pattern of Emx2 (Fig 5C–5E). Second, we quantified hair cell innervation in Vangl2 mutants and found that, as in wild-type specimens, selective innervation correlated with Emx2 expression (Fig 5F–5K) and that approximately half of the hair cell population was stably innervated by each axon (Fig 5L–5O). Together, these data reveal that Emx2 expression correlates with synaptic partnership and, crucially, that selective synaptogenesis is separable from coherent planar polarity.

### Synaptic partnership is determined by Emx2

To test the function of Emx2 in synaptic partnership, we used clustered regularly interspaced short palindromic repeats/CRISPR-associated protein 9 (CRISPR/Cas9)-mediated genome editing to mutate Emx2. Specifically, to facilitate this analysis by live imaging in zebrafish carrying multiple transgenes, we used the CRISPant approach for somatic mutagenesis [47] [48]. We first corroborated that Emx2 loss-of-function renders nearly every hair cell identically oriented, always dorsally in vertical neuromasts (Fig 5P) and rostrally in horizontal neuromasts (Fig 5Q) [44]. Second, we found that in Emx2-deficient animals, individually marked axons synapsed with nearly every hair cell in neuromasts (Fig 5L and 5R–5T) or with a small minority to none (Fig 5L and 5U–5W). Some specimens presented partial expressivity of the Emx2 mutant phenotype, with neuromasts bearing a majority of hair cells oriented in one direction and a small minority in the opposite. Yet in these cases, individually marked axons also synapsed exclusively with hair cells of identical orientation regardless of their absolute or relative number (Fig 5L). These results reveal that loss of Emx2 homogenizes hair cell orientation without altering the ability of axons to recognize identically oriented hair cells and, importantly, that the Emx2 expression status in hair cells determines whether they will be innervated by a specific axon.

## Discussion

The piscine lateral line is a prime example of a neural system that represents in the brain the entire mechanosensory scene as vectorial signals from multiple peripheral receptors [2, 8, 9]. Therefore, it is ideally suited to unravel how neuronal tuning to mechanical-flow direction is established during development and maintained in the face of synaptic instability driven by constant receptor cell turnover. Here, we uncover a simple partner-recognition mechanism that governs the strict innervation of identically plane-polarized hair cells by afferent neurons. The underlying process is robust to environmental perturbations, enabling the maintenance of labeled lines of directional mechanosensory flow in a system that undergoes frequent and life-long renewal of receptor cells. Below, we illustrate this mechanism and discuss the supporting evidence.

### Hair cell projections are dispensable for synaptic selectivity

Similarly to the mammalian utricular and saccular maculae, the hair cells in piscine neuromasts are divided into 2 populations with opposing planar orientation [11, 21]. Utricular and saccular afferent neurons do not co-innervate hair cells across the line of polarity reversal [49–51]. Identical observations have been made in the vestibular apparatus of amphibians [51] and birds [52]. Several studies have revealed that the afferent neuronal pathway of the lateral line is organized in a similar manner [8, 22, 23, 33, 52]. This is remarkable given the absence of anatomical compartmentalization of epithelial planar polarity in neuromasts. To shed light on the synaptic selectivity mechanisms, we experimentally tested the scaffold model. We show that hair cell basal projections are dispensable for polarity-selective innervation, suggesting that they act as haptotactic cues that facilitate or accelerate the innervation of nascent hair cells in a nonselective manner, possibly by increasing the cell’s surface area during the search-and-find period that precedes synaptic maturation [22, 53].

### Planar polarity and synaptic selectivity are separable

The above results raise 4 interwoven questions. What is the source of the selectivity cues? Do selectivity cues act locally in each neuromast or globally along the entire lateral line system? Does a synaptic selectivity code exist? If so, what is its identity? We investigated whether planar polarity per se represents a selectivity cue by using mutant zebrafish lacking the core planar-polarity component Vangl2, in which hair cells assume random orientations. We reasoned that if coherent planar polarity instructed selectivity, its loss would randomize or destabilize synapses. In *Vangl2−/−* animals, connectivity patterns are stable and nonrandom because, as in wild-type specimens, each LAN axon synapses with one-half of the hair cells. Furthermore, our analysis of de novo synaptogenesis by regenerating axons shows a remarkable robustness in the lateral line system in that axons invariably re-innervate hair cells of the same orientation as their original. This remains true even when regenerating axons target a neuromast different from the original. We also found a near-perfect correlation of synaptogenesis when regenerating axons re-innervate neuromasts of differing global orientation because LANs that innervate rcHCs in horizontal neuromasts synapse with dvHCs when switching to innervate vertical neuromasts. Together, these results demonstrate that synaptic selectivity is separable from the local and global orientation of the hair cells and that it is determined independently in each neuromast. It is tempting to extrapolate these results to mammals to explain the puzzling observation that, while the core planar-polarity proteins Frizzled6 and Prikle-like2 distribute identically in the entire epithelium of the sacculus and the utricle, neurons never co-innervate hair cells across the line of polarity reversal [19, 51, 54]. Therefore, it is likely that both in the mammalian inner ear and in the piscine lateral line, planar polarity per se does not determine polarity-selective synaptogenesis. Assessing hair cell innervation in the vestibular system of Vangl2 mutant mice may directly test the likelihood of such evolutionary conservation.

### Emx2 governs the formation and homeostasis of synaptic partnership

The above results suggest the existence of a label that identifies as same caudal-oriented and ventral-oriented hair cells, as well as rostral- and dorsal-oriented cells. They also indicate that the process of planar-polarity–selective synaptogenesis can be framed as a problem of synaptic partner matching rather than of synaptic fidelity. The transcription factor Emx2 is expressed in half of the hair cells in each neuromast and correlates with their global orientation in that Emx2 is exclusively expressed in rcHCs and dvHCs. We found that Emx2 expression patterns are not affected by the loss of coherent planar polarity and that axons make strict connections according to the hair cells’ Emx2 expression status, even when they are not coherently oriented. Moreover, we show that loss of Emx2 affects hair cell innervation in a predictable manner because it enables some axons to synapse with nearly every hair cell in the neuromast while remaining selective as well as simultaneously prevents other axons from making any synapse. These findings demonstrate that Emx2 establishes an identity code for synaptic partners and represents the link between planar polarity and synaptic selectivity. It transpires that there must be differences between the neurons that innervate each hair cell polarity class because, if neurons were identical, regenerating axons should not be able to regain directional tuning by recognizing previous partners or re-innervate matching populations of hair cells when switching from horizontal to vertical neuromasts. Although we currently lack the tools to directly test this hypothesis, we believe that it is likely given some evidence from the mouse, in which a common pool of Neurogenin-1(+) neuronal progenitors produces auditory afferent neurons with segregated connectivity in the inner ear [55].

We wonder how a transcription factor may mediate polarity-selective synaptic connectivity. Genetic perturbation experiments indicate that Emx2 does not control local or global planar polarity because the global axis of polarization of the hair cells is not altered in the inner ear of mice and in neuromasts of zebrafish lacking Emx2. Instead, we interpret the collective data as suggesting that Emx2 instruct hair cell identity, which in turn enables cells to implement the global polarity cue in one of the two possible directions. One possibility is that Emx2-dependent expression of high-affinity complementary transmembrane proteins determines synaptic partnership between matching subpopulations of LANs and hair cells. This will enable neurons to recognize synaptic partners locally and independently of their relative of absolute number and to regain directional tuning after recovery from injury. It also explains how individual neurons synapse with hair cells of differing global orientation when they co-innervate multiple neuromasts during the expansion of the lateral line in juvenile and adult zebrafish [56, 57]. This can occur because synaptic partnership is controlled locally by Emx2, independently of the specific orientation of the hair cells. Taken together, our data strongly support the conclusion that Emx2 establishes an identity code that governs synaptic partnership between hair cells and afferent neurons.

### A wiring algorithm controlling synaptic partnership

**Fig 6:**
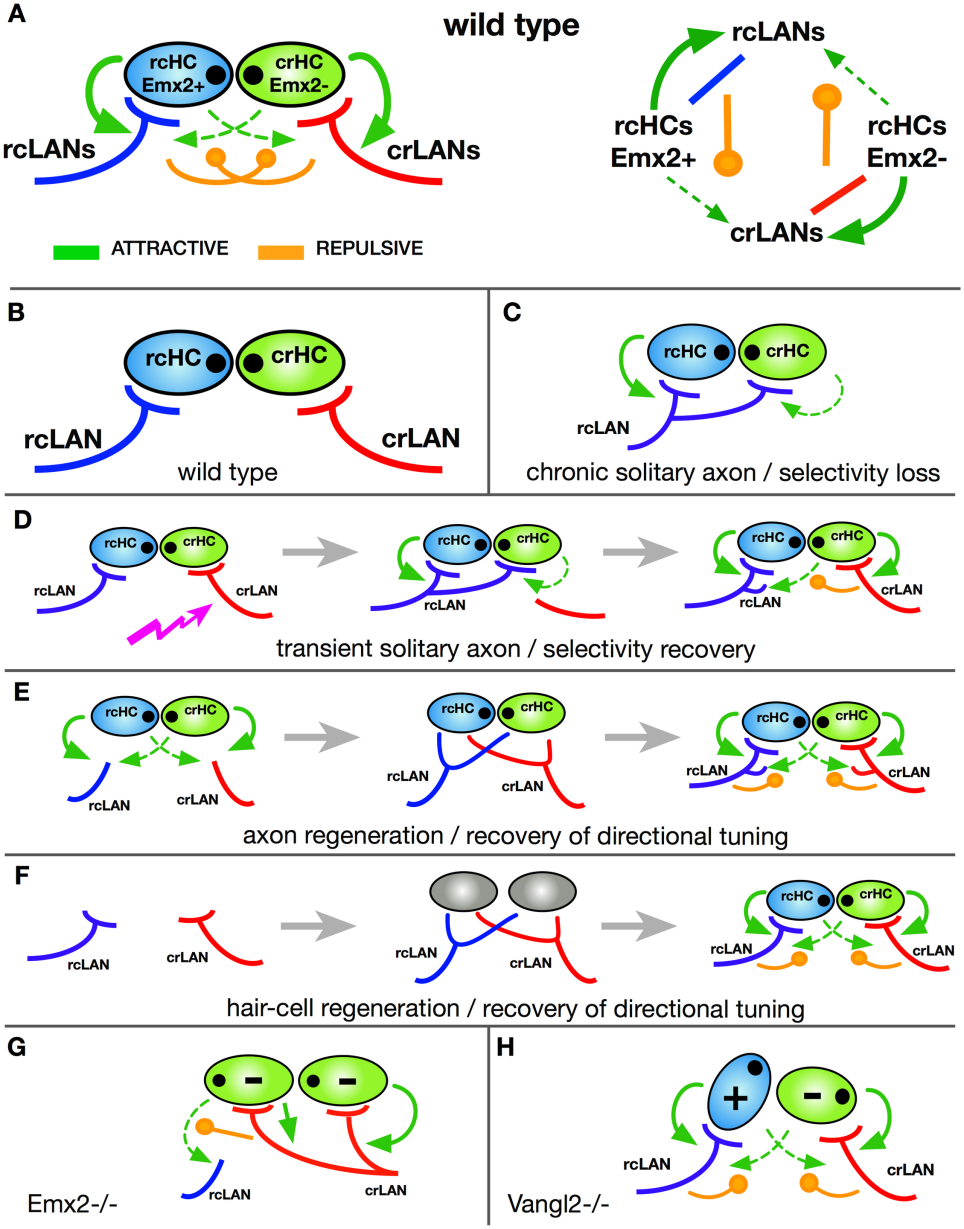
Wiring algorithm underlying the development, homeostasis, and regeneration of planar-polarity–selective synaptogenesis and neuronal directional tuning. (A) Left, schematic description of the wiring algorithm using a wild-type horizontal neuromast as an example. In this and all subsequent panels, cells are color-coded: rcHCs/Emx2(+) (light blue), crHCs/Emx2(-) (light green); eccentric black dots indicate hair cell orientation. rcLANs (dark blue), crLANs (red). Arrows (green) indicate attractive interactions (stronger as solid lines and weaker as dotted lines), and circle-pointed lines (orange) indicate repulsive interactions. Right, schematic description of interactions between cells. (B) Scheme of the constituent cells. (C) Scheme of a situation of a chronically solitary axon, which becomes nonselective because the absence of the converse synapses eliminate the repulsive signals. (D) Scheme of an acutely solitary axon, which becomes transiently nonselective because the regeneration of the converse synapses recreates the repulsive signals to push the promiscuous axon to selectivity. Jagged arrow (purple) indicates axon damage. (E) Situation of axon damage and regeneration. Initially during regeneration, axons explore the presynaptic field and transiently interact with hair cells, but eventually the wiring algorithm is recreated following the same rules controlling selectivity during circuit development. (F) Situation of hair cell loss and regeneration. Initially during hair cell regeneration, axons explore the presynaptic field and transiently interact with hair cells, but once hair cells mature, the wiring algorithm is recreated following the same rules controlling selectivity during circuit development. (G) Situation of homogeneously oriented hair cell upon loss of Emx2 activity. The wiring algorithm becomes asymmetric, directing one axonal population to synapses with every hair cell and inhibiting the other axons from establishing any synapse. (H) Situation of misoriented hair cell upon loss of Vangl2. The wiring algorithm remains normal because it operates independently of coherent planar polarity. Therefore, synaptic selectivity for Emx2(+) and Emx2(-) hair cells occurs normally. crHC, caudorostral HC; crLAN, caudorostral LAN; HC, hair cell; LAN, lateralis afferent neuron; rcHC, rostrocaudal HC; rcLAN, rostrocaudal LAN; Vangl2, van gogh-like 2.

How may the rules governing synaptic partner choice be implemented? In other words, what is the underlying wiring algorithm [58]? Our data suggest a simple structure for the interaction among synaptic partners that can explain the development of planar-polarity–selective synapses, the regeneration of neuronal directional tuning, and the long-term maintenance of labeled lines of directional mechanosensation (Fig 6A).

Crucially, it also unifies all previous results leading to the biased-selector and the scaffold models [32, 33]. The components of the selectivity process are 2 subpopulations of hair cells and t2wo subpopulations of LANs (Fig 6B). In hair cells, this assumption is supported by polarity-associated expression of Emx2 as well as Tmc2b [59]. Hair cells attract axons nonselectively [32]. However, the Emx2 expression status in hair cells determines the outcome of this attraction because synapses will preferentially form when the appropriate axonal subclass contacts hair cells in an Emx2-matching manner. Once formed, stable synapses repulse incorrect partnerships (Fig 6A). This explains the promiscuous synaptogenesis by solitary axons, which become doubly tuned because the absence of other axons prevents the formation of the converse correct synapses, eliminating the repulsive signals (Fig 6C). A combination of attractive and repulsive signals also explains the recovery of selectivity by the reintroduction of additional axons, which reestablish synapses with the correct partners based on Emx2 expression status, regenerating the repulsive signal that eventually pushes the promiscuous solitary axon to exclusively synapse with hair cells of identical polarity (Fig 6D) [32].

Previous work has shown that chronic loss of hair cell innervation does not affect epithelial planar polarity in neuromasts. This has led to the prediction that innervation does not determine Emx2 expression patterns, which we have now confirmed experimentally. This explains how regenerating axons are able to synapse with the original hair cells (Fig 6E). It occurs because Emx2 expression in denervated hair cells does not change, thus serving as an indelible mark of previous partnership. Our model also explains the recovery of selectivity and neuronal directional tuning after hair cell death and regeneration because the system continuously self-organizes by reenacting the developmental program that assigns different identities to sibling hair cells, in turn determining the likelihood of synaptic stabilization of specific axons (Fig 6F). Upon loss of Emx2, the entire hair cell population becomes homogeneously oriented [44]. In this case, the architecture of the wiring algorithm becomes asymmetric, directing one axonal population to synapses with every hair cell and inhibiting the other axonal population from establishing any synapse (Fig 6G). Furthermore, because loss of mechanoreception does not affect hair cell planar polarity, hair cell activity cannot affect Emx2 expression, explaining why synaptic selectivity is normal in fish with nonfunctional hair cells [23, 32]. Finally, selective synaptogenesis is maintained in the absence of coherent epithelial planar polarity because Emx2 remains expressed in half of the hair cells in Vangl2 mutants (Fig 6H), and thus the architecture of the algorithm remains intact (Fig 6A).

## Conclusion

Three questions in mechanosensory biology are the current focus of intense attention. First, what is the mechanism that establishes the connectivity patterns between distinct mechanoreceptors and ascending afferent neurons [1, 32, 60]? Second, how are the resulting labeled lines of directional mechanosensation maintained in the face of continuous turnover of receptor cells [1, 32, 60]? Third, how much has the anatomical blueprint for directional mechanosensation diverged during the 600 million years of evolution since hair cell–based mechanosensation appeared in the common ancestor of agnathans and mammals [50, 54, 61, 62]? We found that selective connectivity between LANs and hair cells of identical orientation occurs independently of presynaptic projections and that it is separable from the coherent planar polarity and global orientation of the hair cells. We demonstrate a wiring algorithm that locally disambiguates hair cell identity, links identity to orientation, and establishes the synaptic partnership rules that generate neuronal directional tuning. Despite its simplicity, this algorithm readily solves the selectivity task even in the presence of constant noise generated by frequent death and regeneration of hair cells, and independently of scale differences between the receptive organ and the neuronal population. It also operates robustly upon evolutionary changes in epithelial polarity, or when neurons co-innervate organs with differing global orientation, which naturally occurs during the expansion of the lateral line that accompanies animal growth [56, 57]. The striking similarities between the zebrafish lateral line and the mammalian vestibular apparatus, including their dependence on Emx2 for hair cell orientation, suggest that the wiring mechanism that we have unveiled may be ancient and conserved across vertebrates.

## Materials and methods

### Ethics statement

Experiments with wild-type, mutant, and transgenic embryos of undetermined sex were conducted under a protocol approved by the Ethical Committee of Animal Experimentation of the Parc de Recerca Biomedica de Barcelona (Spain), and protocol number Gz.:55.2-1-54-2532-202-2014 by the “Regierung von Oberbayern” (Germany).

### Zebrafish strains and husbandry

Naturally spawned zebrafish eggs were collected and cleaned, and embryos were maintained in system water under standardized conditions at 28.5 °C, at a maximum density of 50 individuals per 85-mm Petri dish. The transgenic and mutant lines used in this study have been previously described: Tg[pou4f3:GAP-GFP] [38], Tg[pou4f3:ctbp2l-mKOFP] [26], Tg[myo6b:actb1-EGFP] [40], Tg[hsp70l:mCherry-2.0cntnap2a] [32], Tg[nkhgn39d] [22], and Et(krt4:EGFP)sqet4 [46]. The neurogenin1 mutant line was neurog1^hi1059^ [32], and the trilobite/Vangl2 mutant was Df(Chr07:stbm)^vu7^ [45, 46].

### DNA constructs

The hsp70:mCherry-SILL (SILL:mCherry) construct was generated using the Tol2 kit. Entry vectors were generated as described in the Invitrogen Multisite Gateway manual. PCRs were performed using primers to add att sites onto the end of DNA fragments, using Platinum Pfx (Invitrogen). The pEntry vectors containing the UAS sequence, hsp70 minimal promoter, mCherry, and polyA are from the Tol2 kit, and the pEntry vector containing the SILL enhancer was previously generated in our laboratory [32].

### Mosaic neuronal labeling

For sparse labeling of LANs, we injected 15 to 20 pg of a DNA plasmid containing the construct hsp70:mCherry-SILL (SILL:mCherry) in 1– to 4–cell-stage embryos. Resulting embryos at 3 to 4 dpf were anaesthetized and screened for red fluorescence in single neurons using a Zeiss stereomicroscope.

### Emx2 loss-of-function

Targeted somatic mutagenesis of Emx2 was done using the CRISPant strategy [47]. To this end, a solution containing sgRNA (160 ng/μl), Cas9 (760 ng/μl), and SILL:mCherry DNA (20 ng/μl) was incubated for 5 minutes at 37 °C and injected in 1–cell-stage eggs of the transgenic lines Tg[Myo6b:actb1-EGFP; pou4f3:ctbp2l-mKOFP] and Tg[myo6b:actb1-EGFP]. sgRNA was designed using the online tool CCTop—Crispr/Cas9 target online predictor (crispr.cos.uni-heidelberg.de). The selected target sequence used in this study was GGAGGAGGTACTGAATGGACTGG.

### Immunohistochemistry

Larvae were fixed overnight at 4 °C in a solution of 4% PFA. After fixation, fish were washed with PBST and permeabilized in acetone at −20 °C for 5 minutes. Then, samples were washed with MiliQ water for 5 minutes and incubated for 1 to 2 hours at room temperature with blocking solution (1% BSA, 2% NGS, 1% DMSO). After blocking, larvae were incubated overnight with primary Ab (Ctbp2 1:100) or for 48 hours (Emx2 1:250) at 4 °C. The next day, fish were washed with PBST 5 to 6 times and incubated overnight at 4 °C with secondary Ab (GaRb 633, GaRb 555). Then, samples were washed with PBST and mounted for imaging. The Ctbp2 primary antibody was obtained from Proteintech (Manchester, UK) and the Emx2 antibody from TransGenic (Fukuoka, Japan).

### Imaging and videomicroscopy

For in vivo imaging, laser-mediated axotomy, and some Emx2 immunostaining, we used a custom-built inverted spinning disc microscope (Zeiss Axioscope). Emx2 immunostainings in wild type with single neurons labeled were imaged using a Zeiss inverted confocal microscope with a 40× water immersion objective. Embryos and larvae used for in vivo imaging were anesthetized in MS-222 (tricaine) 0.16 g/L and mounted in 1% low–melting-point agarose on the cover slip of a glass-bottom dish (MatTek, Ashland, MA). Imaging dishes were bathed in Danieau’s with MS-222 0.16 g/L, except for time-lapse imaging where concentration was reduced to 0.08 g/L. Acquisition was performed at 28.5 °C using a 63× water immersion objective.

### Assessing synaptic connectivity

Synaptic connectivity was assessed in transgenic fish expressing different fluorescent markers in all hair cells and in individual axons. Synapses were identified as bulged postsynaptic endings adjacent to the base of hair cells. This was always done by progressing through individual focal planes of Z-stacks, from the apical end of the epithelium (to assess the planar polarization of each hair cell) to the most basal aspect of the epithelium (where apposition of neuronal endings and hair cells are found). Examples are shown in 2 supplemental videos (S3 and S4 Videos, associated with Fig 3).

### Statistical analyses

The total number of hair cells is not uniform across different neuromasts. Thus, quantification of the innervation in wild-type, Vangl2, and Emx2 mutant specimens was normalized to the total number of hair cells in each neuromast. We compared the obtained fractions in the different groups using an ANOVA test. For the analysis of Emx2-positive cells in wild-type and Vangl2 mutants, we also normalized samples by dividing the number of positive hair cells by the total number of hair cells. We then compared the wild-type and Vangl2 mutant groups using an unpaired *t* test.

### Neomycin treatment and laser-mediated microsurgery

Specimens were incubated in MS-222 (3-aminobenzoic acid ethyl ester) in E3 medium (5 mM NaCl, 0.17 mM KCl, 0.33 mM CaCl2, and 0.33 mM MgSO4, in deionized water [pH 7]) and mounted in 1% low-melting agarose on a glass-bottom Petri dish. They were positioned using a hair loop under light from a xenon arc lamp passed through the appropriate filters to reveal green or red fluorescence. Axon severing wad done using a computer-controlled iLas-Pulse laser system (Roper Scientific SAS, Evry, France) consisting of a pulsed ultraviolet laser (355 nm; 400 ps/2.5 μJ per pulse). The axons were transected with a focused laser beam coupled to a spinning-disk inverted microscope. The laser power was set to 35 mW at the sample plane. To sever axons, a region of interest (ROI) was drawn over the nerve, and a train of laser pulses was repeatedly applied until all fluorescence disappeared within the ROI. After severing, the axons were observed repeatedly to evaluate the success of the cuts. Subsequently, specimens were removed from the agarose and left to recover in fresh E3 embryo medium in individual Petri dishes. For regeneration experiments, larvae are remounted as the above for time-lapse imaging. Z-stacks were made using ImageJ (Plugins → Segmentation → Simple Neurite Tracer). Hair cells were pharmacologically ablated by incubation of specimens in a 250 μM solution of neomycin for 45 to 60 minutes at room temperature and then rinsed with E3 medium [26, 39, 46].

## Supporting information

S1 Video Developmental innervation of hair cells by a singly marked axon. This video shows an example of the innervation of hair cells expressing EGFP (green) as they develop, by a single axon marked by mosaic expression of mCherry (red). Hair cells also express the active-zone component Ribeye fused to Kusabira (orange). Basal projections from hair cells are readily evident during the first half of the video. Yet Ribeye is never detected in the projections, and synaptic stability occurs by the juxtaposition of LAN neuritis and Ribeye-containing puncta in the basal aspect of hair cells that do not produce projections. EGFP, enhanced green fluorescent protein; LAN, lateralis afferent neuron.
(AVI)

S2 Video Regenerative innervation of hair cells by a singly marked axon. This video shows an example of the re-innervation of mature hair cells expressing EGFP (green) by a previously severed single axon marked by mosaic expression of mCherry (red). Basal projections are not produced by these hair cells. Yet synaptogenesis occurs normally. Thin green extensions towards the end of the video are apical kinocilia from the hair cells, which become evident due to their free movement. EGFP, enhanced green fluorescent protein.
(AVI)

S3 Video Determination of synapses in a horizontal neuromast by individualized axon before severing. This video is a Z-stack used to generate panel P–P’ of Fig 3 and shows an example of the innervation of mature hair cells expressing EGFP (green) by an axon marked by mosaic expression of mCherry (red). Red dots indicate the synapsed hair cells, whose planar polarization is evident in the most apical aspect of the epithelium (beginning of the video), revealing their caudorostral orientation (as depicted in Fig 3Q). Yellow arrows indicate synaptic contacts as bulged axon endings. EGFP, enhanced green fluorescent protein.
(AVI)

S4 Video Determination of synapses by the individualized axon that initially innervated the horizontal neuromast, after severing and regeneration to innervate a vertical neuromast. This video is a Z-stack used to generate panel R–R’ of Fig 3 and shows an example of the re-innervation of mature hair cells expressing EGFP (green) by an axon marked by mosaic expression of mCherry (red). Red dots indicate the synapsed hair cells, whose planar polarization is evident in the most apical aspect of the epithelium (beginning of the video), revealing their ventrodorsal orientation (as depicted in Fig 3S). EGFP, enhanced green fluorescent protein.
(AVI)

S5 Video Assessment of Emx2 immunostaining. This video shows an example of a Z-stack of a neuromast immunostained for Emx2. White circles mark 5 Emx2(+) hair cells (magenta). Numbers reflect order of appearance across the Z-stack from the epithelial apex to base. This Z-stack was used to generate the maximal-projection image shown in Fig 4K and is exemplified in drawing of Fig 4L.
(AVI)

S1 Table Numerical data for the plots in Fig 5. This table contains the data point used for statistical tests plotted in Fig 5E (left) and Fig 5L (right), including all conditions that include wild-type and mutant specimens.
(TIFF)

## Abbreviations

crHC: caudorostral HC
CRISPR/Cas9: clustered regularly interspaced short palindromic repeats/CRISPR-associated protein 9
DiAsp: 4-(4-diethy-laminostyryl)-N-methylpyridinium iodide
dvHC: dorsoventral HC
EGFP: enhanced green fluorescent protein
HC: hair cell
LAN: lateralis afferent neuron
rcHC: rostrocaudal HC
ROI: region of interest
Vangl2: van gogh-like 2
vdHC: ventrodorsal hair cell
