# Supplementary figures and images for: Hair cell identity establishes labeled lines of directional mechanosensation
Source: PLoS Biol. 2018 Jul 19;16(7):e2004404. doi: 10.1371/journal.pbio.2004404 (PMC6067750; doi:10.1371/journal.pbio.2004404)

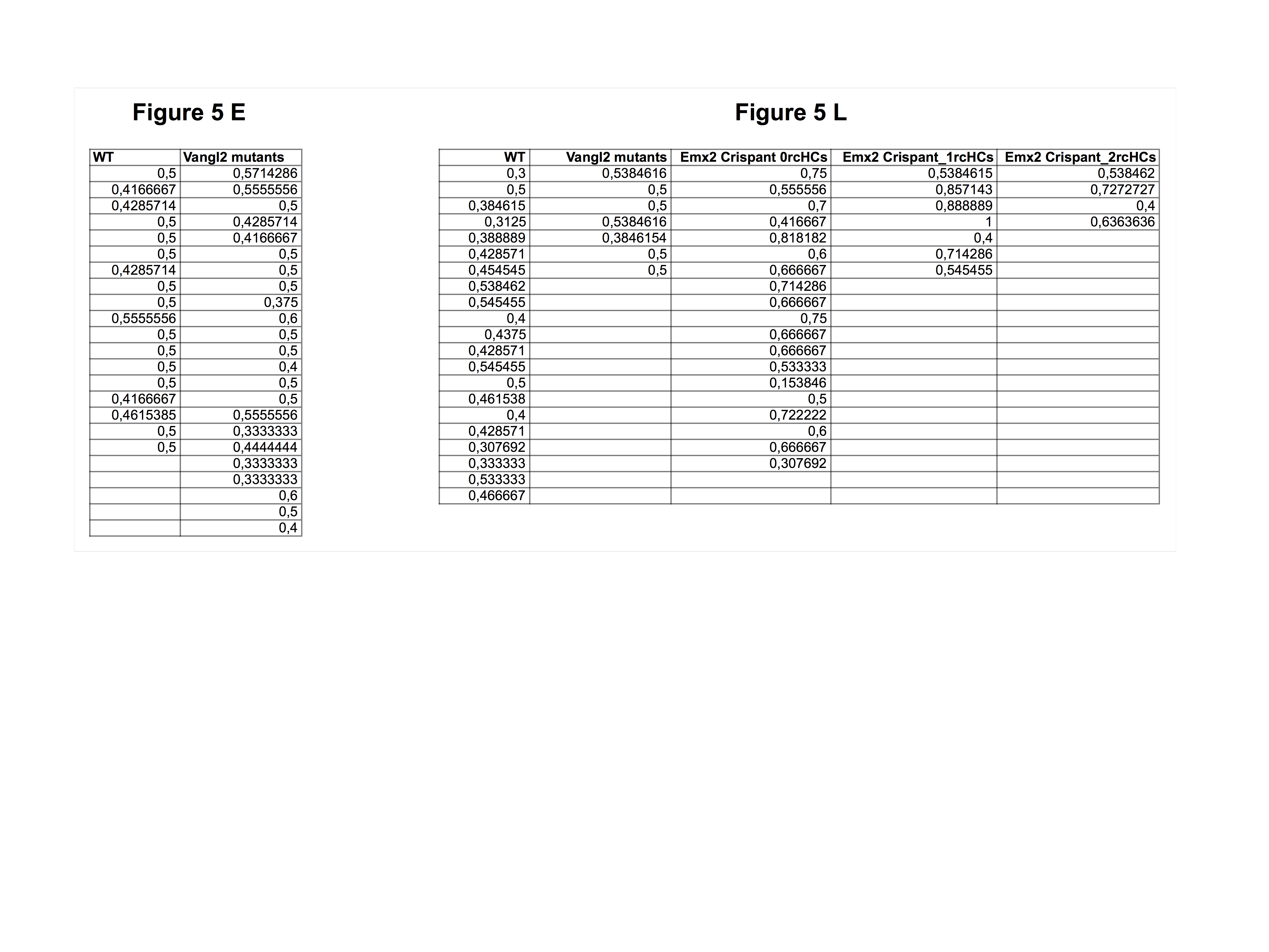

Supplement: S1 Table — This table contains the data point used for statistical tests plotted in Fig 5E (left) and Fig 5L (right), including all conditions that include wild-type and mutant specimens. (TIFF) [file pbio.2004404.s006.tiff]
